# Time perception at different EEG-vigilance levels

**DOI:** 10.1186/1744-9081-8-50

**Published:** 2012-09-21

**Authors:** Juliane Minkwitz, Maja U Trenner, Christian Sander, Sebastian Olbrich, Abigail J Sheldrick, Ulrich Hegerl, Hubertus Himmerich

**Affiliations:** 1 Department of Psychiatry and Psychotherapy, University of Leipzig, Semmelweisstr. 10, 04103, Leipzig, Germany; 2 Leipzig University Medical Center, IFB Adiposity Diseases, Stephanstr. 9c, 04103, Leipzig, Germany; 3 Department of Psychiatry and Psychotherapy, University of Rostock, Gehlsheimer Str. 20, 18147, Rostock, Germany; 4 Claussen-Simon-Endowed Professorship for Neurobiology of Affective Disorders, Department of Psychiatry and Psychotherapy, University of Leipzig, Semmelweisstr. 10, 04103, Leipzig, Germany

**Keywords:** Electroencephalography (EEG), Time perception, Vigilance, Vigilance Algorithm Leipzig (VIGALL)

## Abstract

**Background:**

Human time perception is influenced by various factors such as attention and drowsiness. Nevertheless, the impact of cerebral vigilance fluctuations on temporal perception has not been sufficiently explored. We assumed that the state of vigilance ascertained by electroencephalography (EEG) during the perception of a given auditory rhythm would influence its reproduction. Thus, we hypothesised that the re-tapping interval length and the accuracy of reproduction performance would vary depending on the state of vigilance determined by EEG.

**Methods:**

12 female and 9 male subjects ranging from 21 to 38 years (M = 25.52, SD = 3.75) participated in a test paradigm comprising a) a resting EEG for the determination of vigilance while an auditory rhythm was presented, b) a short activity of the proband to be sure of sufficient alertness, and c) a tapping task to reproduce the presented rhythm. Vigilance states of three consecutive 1-sec-EEG-segments of the resting EEG before the reproduction phase were classified using the Vigilance Algorithm Leipzig (VIGALL).

**Results and discussion:**

Reproduction accuracy was more precise after high EEG-vigilance stages. Thus, the subjects’ mean deviation from the given rhythm was lower (t(17) = −2.733, p < 0.05) after high vigilance stage A (MW = 0.046, SD = 0.049) than after low vigilance stage B (MW = 0.065, SD = 0.067). The re-tapping-length was significantly shorter (t(17) = −2.190, p < 0.05) for reproduction phases following high EEG-vigilance stage A compared to the lower EEG-vigilance stage B.

**Conclusion:**

These findings support the hypothesis of a varying time perception and of speed alterations of the internal clock after different states of EEG-vigilance, which were automatically classified by VIGALL. Thus, alterations of cognitive processing may be assessable by specific EEG-patterns.

## Introduction

The human perception of the rate time passes in is rather instable as can be demonstrated in a number of settings. Alertness and excitement can considerably influence individual time perception. For instance, while awaiting an intensely anticipated event, time is perceived to pass rather slowly. Also, time is perceived to decelerate in tedious situations such as afternoon school lessons. The goal of this study is to verify the validity of these subjective observations by investigating the influence of objectively classified states of wakefulness on the subjective sense of time.

The investigation of alterations in time perception has been a subject of research for decades. There is extensive literature on the topic of how and where in the brain time is processed [1]. Furthermore, several time modulating factors, such as personality, attention and emotions have been described in the literature [2]. A separate branch of research focuses primarily on time processing. However so far, previous studies have not yielded an entirely accepted model [3-7].

Several studies examining patients suffering from neurological disorders such as brain lesions [8,9], stroke [10] and Parkinson’s disease [11,12] provide evidence that specific brain areas are linked to time processing. Moreover, data from neuroimaging studies support the importance of these brain areas for time perception and processing [13-16].

In order to capture of the subjective passing of time, two main standard tasks have been established: Time production tasks ask subjects to push a button if they think that a certain time span has already passed. Time estimation tasks request subjects to evaluate the length of previous time spans by timing seconds or minutes.

Prior publications suggest that individual time perception differs between individuals (inter-individual variability) and also within a single person (intra-individual variability), depending on certain conditions or individual states [17,18]. Particularly, the influence of fluctuations of diurnal individual arousal [19,20] is discussed in the literature concerning time perception variations. Thus, a conceivable approach to investigate individual alterations of time perception might be to determine individual states of wakefulness. However, the literature provides overlapping wakefulness concepts and terms: e.g. alertness, arousal and vigilance [21]. The vigilance concept we refer to in our study describes unspecific activation states of the central nervous system on the sleep-wake spectrum as they are empirically assessable by EEG. Loomis et al. [22] classified different activation states of the brain on a continuum reaching from the concentrated awake state to the state of deep sleep on the basis of specific EEG-patterns. These EEG-vigilance stages have been subdivided (A1, A2, A3, B1, B2/3) by Bente [23] and Roth [24] in dependence on the frequency and topographic distribution of EEG-waves. For detailed description of the EEG-vigilance stages, see Additional file 1: Table S1. By determining the individual state of EEG-vigilance and measuring the performance on standard timing tasks, conclusions on the influence of vigilance on time perception could be drawn.

Due to methodological difficulties, EEG-vigilance stages have not been examined on single-trial basis to date. Moreover, even expert raters showed poor performance in identifying vigilance lapses using EEG [25]. Therefore, a computer-based algorithm (Vigilance Algorithm Leipzig, VIGALL) was developed by Hegerl et al. [26]. VIGALL classifies different EEG-vigilance stages (A1, A2, A3, B1, B2/3) of EEG segments, according to Bente [23] and Roth [24], on the basis of the frequency and topographical distribution of neuroelectric activity. Olbrich et al. [27] validated the algorithm taking simultaneous recordings of EEG and functional magnetic resonance imaging (fMRI) data into account. VIGALL was further improved by including EEG-power source estimates using sLORETA (standardized Low Resolution Brain Electromagnetic Tomography). Additional file 1: Table S1 depicts decision criteria of the algorithm to calculate vigilance stages from the obtained EEG data.

Overall, the main purpose of the present study was to examine the influence of the EEG-based vigilance level on time perception using the VIGALL. In our study, we adhere to the classic internal clock model [5]. We expect that a high level of vigilance leads to a slowing down of the subjective passing of time. Thus, the internal clock might produce more time units (analogous to the ticks of a clock) in shorter time intervals. By contrast, we assume that fewer time units are produced by the internal clock in low vigilance states implicating an acceleration of the subjective passing of time.

For assessing the individual sense of time, we applied a tapping paradigm according to findings of previous studies, which provide evidence that the tapping speed correlates with the speed of the internal clock [4]. Thus, we assume that the re-tapping speed increases significantly after low EEG-vigilance levels, as external stimuli are subjectively perceived to be accelerated (Additional file 2: Table S2). Vice versa, we expect a decrease of the re-tapping speed after high EEG-vigilance states. We additionally hypothesize that the vigilance level has a crucial impact on the accuracy the perceived rhythm is reproduced with.

## Material and methods

### Participants

In total, 21 subjects ranging from 21 to 38 years (M = 25.52, SD = 3.75) participated in the study. The sample consisted of 12 female and 9 male participants not differing significantly in age (p = 0.884). Volunteers were recruited through advertisement and received 15€ remuneration. All participants were free of any psychiatric, neurological or other serious medical condition. Physical health was screened in a semi-structured interview; mental health was examined according to criteria of the Diagnostic and Statistical Manual of Mental Disorders (DSM-IV) by applying a German version of the Structured Clinical Interview for DSM-IV disorders (SKID) [28]. To exclude drug and alcohol abusing subjects, general alcohol and drug consumption was quantified by exerting the Alcohol Use Disorders Identification Test (AUDIT) [29] and the Drug Use Disorders Identification Test (DUDIT) [30]. The local ethics committee approved the study. Written informed consent was obtained from each volunteer prior to investigation according to the declaration of Helsinki.

### Measures and procedures

To avoid circadian effects, EEGs were recorded at 7 am in a dimmed and sound attenuated room. To assure vigilance decline, all subjects worked the night before. EEG recording took place directly after work had finished. Resting EEG recordings were conducted with closed eyes in a half-lying position during the tapping task. The testing procedure took about 60 minutes. The VIGALL algorithm was used to classify EEG-vigilance.

### Tapping paradigm

EEG was performed with closed eyes in a horizontal position. The tapping paradigm used in this study contained several trials consisting of a presentation phase, a short activation of the subject (by an acoustic signal) and a reproduction phase. During the presentation phase, an auditory rhythm was presented, which had to be re-tapped in the reproduction phase. Subjects were activated between presentation and reproduction by an acoustic signal. EEG data were recorded during the presentation phase.

To encourage a decrease in vigilance, subjects were not given any task during the presentation of the auditory rhythm. The auditory stimulus was consistent in pitch (350 Hz) and was presented for 200 ms with a constant inter-stimulus-interval of 800 ms. Nevertheless, subjects were informed by the instructor that the rhythm of the tones alters within a small range to assure that the subjects did not keep solely the first tone presentation in mind for the reproduction phase.

The auditory rhythm was presented in variable intervals of 30 seconds to 5 minutes. The goal was to gather maximal data variability due to statistical reasons, i.e. each participant should ideally reproduce the rhythm after low vigilance phases as frequent as after high vigilance phases. Therefore, the investigator monitored EEG recording and state of vigilance in order to be able to actively vary the interval length of the presentation phase by initiating an acoustic signal. This preselection concerned the habitual differences of vigilance declines between subjects due to different EEG-vigilance regulation patterns [31].

The acoustic signal (600 Hz, 200 ms) challenged the subjects to re-tap the recently perceived sequence of the auditory stimuli on a touchpad for about 15 seconds. The participants were instructed to push a button quickly in case of when they perceived the acoustic signal to ensure compliance. To divide vigilance between presentation and tapping phase, subjects were further briefed to engage in stretching as a short activity after presentation of an acoustic signal to enhance alertness. Mean inter-tapping-time of each trial was calculated.

The vigilance stages of the last three consecutive 1-sec-EEG-segments of the resting EEG before the reproduction phase were classified using the Vigilance Algorithm Leipzig (VIGALL).

### EEG

EEG recordings lasted about one hour without preparation time. EEG was performed by placing 31 electrodes (sintered silver/silver chloride) according to the extended 10–20 international system. Impedances were kept below 10 kOhm and common average was used for reference. Data was recorded with 1 kHz sampling rate. To control for cardial and ocular artefacts, both electrocardiogram (ECG) and electrooculogram (EOG) were recorded simultaneously. One EOG-electrode was taped on the right forehead and a reference electrode was fixed on the cheek below the right eye. ECG-electrodes were set on the right and left wrist. EEG recordings were carried out with the BrainVision Analyzer 2.0 software (BrainProducts, Gilching, Germany). These were amplified by a 40-channel-QuickAmp unit (BrainProducts, Gilching, Germany).

EEG data was pre-processed with the Analyzer software package. First, low-pass (70 Hz), high-pass (0.5 Hz) and notch filters (50 Hz, range 5 Hz) were used to filter raw data EEG sets. Then, correction of EEG-channels with continuous muscle activity and removal of eye artefacts was performed by using an independent component analysis (ICA)-based approach [32,33]. Afterwards, the data sets were segmented into consecutive one-second intervals and again screened for remaining muscle, movement, eye and sweating artefacts. Segments containing artefacts were excluded from EEG-vigilance stage analysis. To obtain the frequency band envelope magnitude in μV^2^ in order to approximate the power of the underlying signal [34], complex demodulation of the EEG-frequency bands 2-4 Hz (delta), 4-8 Hz (theta), 8-12 Hz (alpha) and 12-25 Hz (beta) was computed for all EEG channels. Thereafter, intracortical averaged squared current densities of frequency band power was calculated in four predefined regions of interests (ROIs) by using the sLORETA module of the Vision Analyzer software:

▪Occipital ROI: occipital lobe and the cuneus, as alpha activity during rest is most prominent in those areas [35].

▪Parietal ROI: superior and inferior parietal lobe, in these areas shifts of alpha power have been found during the transition phase from full wakefulness to sleep [36,37].

▪Temporal ROI: inferior temporal lobe, most prominent EEG-alpha power has been found in the inferior lobe during light sleep stages [38].

▪Frontal ROI: anterior cingulate gyrus (ACC) and the medial frontal gyrus, most prominent EEG alpha power and EEG theta power is located within these areas during drowsiness [39,40].

According to EEG-source estimates in the ROIs, EEG-vigilance stages were classified by the VIGALL algorithm (see Additional file 1: Table S1). Lower vigilance stages, characterised by K-complexes and sleep spindles, did not occur within data sets. For statistical analysis, vigilance stages of the last three consecutive 1-sec-segments of the presentation phase were evaluated. EEG-vigilance sub-stages were subsumed under main EEG-vigilance stages A (A1, A2, A3) and B (B1, B2/3).

### Data preparation

Several EEG recordings had to be terminated prematurely due to an increased proportion of apparent artefacts as a consequence of decreased relaxation of the participant or similar reasons. The number of executed trials during testing procedure varied due to EEG quality and EEG-vigilance regulation patterns [31]. Subjects with rare vigilance switches (stable vigilance regulation patterns) were tested for a longer period to obtain a broad vigilance variance. On average, the procedure contained 25 trials (SD = 7.52) per subject. VIGALL classified the EEG-vigilance levels for the last three seconds of the presentation phase. In case of a stable vigilance state within these three seconds, i.e. three equal VIGALL-vigilance classifications within the three-second-sequence, trials were included for calculating the average vigilance-specific re-tapping interval length per subject. Hereby, we focussed on the last three seconds, as we supposed that the actual vigilance has an influence on the participants’ perception. Besides, trials with tapping standard deviations greater than 1000 ms were treated as missing values as they potentially reflect overt omissions and errors due to e.g. key mal-functions. Trials with response times above 2000 ms were excluded from further analysis for the same reason. Additionally, subjects fulfilled a further inclusion criterion if at least two re-tapping trials per EEG-vigilance stage A and B were available. Thus, three subjects had to be excluded from statistical analyses. 18 (10 female and 8 male) subjects ranging from 21 to 38 years (MW = 25.78, SD = 3.96) remained for statistical comparison between the main EEG-vigilance stages A and B. Detailed analyses between separate sub-stages were not computed because most subjects did not reach each EEG-vigilance sub-stage.

For analysis of re-tapping accuracy, we calculated the absolute value of deviation of the re-tapped rhythm from the given rhythm for EEG-vigilance stage A and B.

### Statistical analyses

All data were processed using the PASW Statistics 18.0 Package for Windows. Tapping performance data from trials were averaged for each EEG-vigilance main stage (A and B). The mean length of the inter-tapping-interval and the mean absolute value of deviation from the presented rhythm was calculated for EEG-vigilance stage A and B for each subject. Participants performed at least two trials (M_A_ = 11.89, SD_A_ = 5.290, M_B_ = 6.78, SD_B_ = 5.298) during each vigilance stage. Paired t-tests were used to assess the vigilance effect on tapping speed and accuracy. Hypotheses were tested one-tailed, the α-level was set to p = 0.05, marginal trends were determined up to significance level 0.10.

## Results

### Vigilance

The relative proportion of the main vigilance stages A and B was determined for each participant. A-stages (M = 64.81%, SD = 21.01%) occurred significantly more frequently (t(17) = 3.283, p < 0.05) than B-stages (M = 35.19%, SD = 21.01%). There were no significant sex differences in the frequency of occurrence of the different stages.

### Tapping speed and vigilance

**Figure 1:**
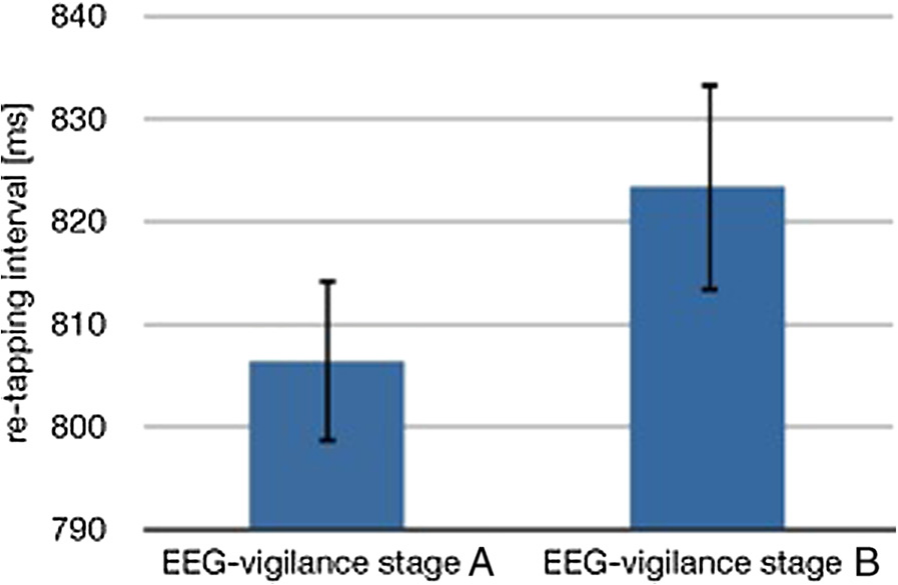
**Mean re-tapping interval length ± standard deviation (SD) for EEG-vigilance stages A and B (N = 18).** Subjects re-tapped significantly (p < 0.05) faster during high EEG-vigilance stage A.

Mean inter-tapping-intervals were calculated for each participant concerning the two main EEG-vigilance conditions (stage A and B). Paired t-tests were used to assess the difference of the tapping speed within the vigilance conditions. When comparing the inter-tapping-intervals of A- (M = 0.806 s, SD = 0.067 s), and B-stages (M = 0.823 s, SD = 0.087 s), the tapping speed (Figure 1) was significantly reduced (t(17) = −2.190, p < 0.05) after a lower level of EEG-vigilance (stage B). The mean inter-tapping-intervals of the A- and B-stages did not differ significantly from the given interval of 800 ms (t_A_(17) = 0.396, p = 0.697; t_B_(17) = 1.137, p = 0.271). No sex differences were observed.

### Tapping accuracy and vigilance

**Figure 2:**
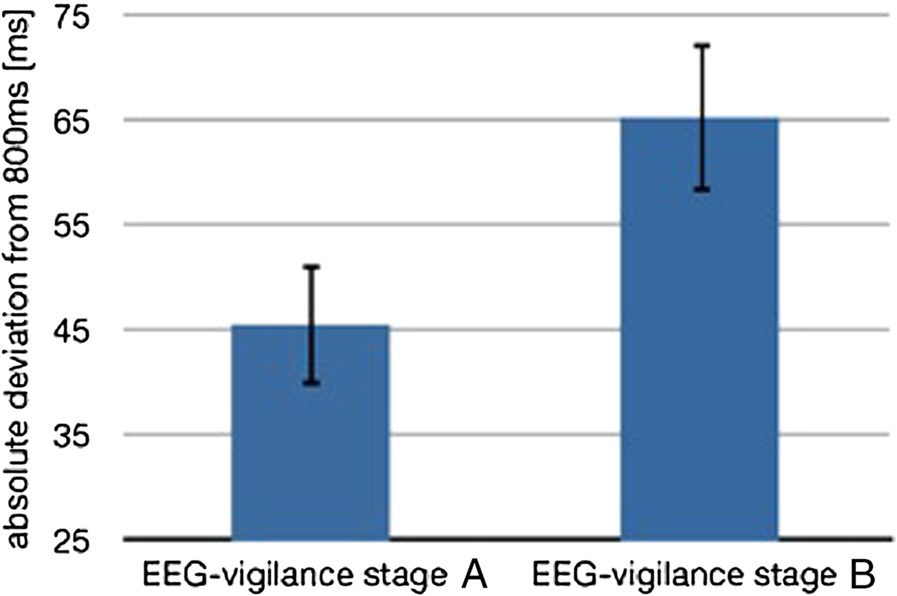
**Mean value of the absolute deviation from the given interval length of 800 ms ± SD (N = 18).** Subjects re-tapped the given rhythm more accurately in case of high EEG-vigilance stage A.

Subjects’ mean deviations from the given rhythm were calculated for A- and B-stages (Figure 2). Vigilance has a significant impact on the accuracy of reproducing rhythms (t(17) = −2.733, p < 0.05) as subjects reproduced rhythms more precisely after higher vigilance stage A (MW = 0.046, SD = 0.049) than after lower vigilance stage B (MW = 0.065, SD = 0.067). There were no significant sex differences in the tapping accuracy in dependence of the vigilance stage.

## Discussion

The aim of our study was to assess the impact of EEG-vigilance on time perception. We revealed that the level of wakefulness significantly influences the temporal perception of external events. However, the direction of the tapping speed effect is contrary to our initial hypothesis. While we assumed that the tapping speed might be enhanced after lower vigilance stage B due to the slowing down of the internal clock and perception of more external events per time unit, subjects tapped faster succeeding high vigilance stage A. According to our accuracy hypothesis, subjects re-tapped the auditory rhythm more accurately after high vigilance stage A than after low vigilance stage B.

A possible explanation for this result which is contrary to our initial hypothesis might be that the difference in vigilance between presentation phase and tapping phase was too small. Subjects who were in a drowsy state during both experimental phases might have tapped slower due to their lower activation level. The acoustic signal and the short activity were possibly not strong enough stimuli to trigger a sustained central nervous activation. Moreover, subjects who were in the high vigilance stage A during the presentation and reproduction phase might have tapped faster owing to an accelerated internal clock. Furthermore, the omitted variability of the auditory rhythm might have affected the subjects’ compliance.

Assuming that the vigilance stage during the presentation of the auditory rhythm is similar to the vigilance stage during the tapping phase, our results are consistent with the outcome of several studies examining the impact of arousal on time perception. A study by Droit-Volet [2] demonstrated that the presentation length of emotional pictures is more likely to be overestimated compared to neutral stimuli due to an increased level of arousal in case of perceiving emotional pictures. Gil et al. [41] reported an acceleration of the internal clock in the case of an enhanced anger-associated arousal. Furthermore, findings of an early study by Anliker [42] concerning the relationship between variations in alpha voltage of the electroencephalogram and time perception are in accordance with our results. By determining the percentage of alpha voltage, states of consciousness were categorized as “very drowsy”, “relaxed”, or “alert”. In contrast to our study, where vigilance stages were determined by the automatic EEG-algorithm VIGALL, vigilance states were classified by simple identification of alpha voltage in this study. More drowsy states caused extended tapping phases, because subjects underestimated the passage of time and thus overproduced time lengths. Werboff [43] reported that basic EEG patterns, which were recorded before the experiment started, predict the tendency to overestimate or underestimate short temporal intervals during the experimental phase. Subjects showing less than 50 per cent alpha waves (low vigilance group) in the closed-eyes condition were compared to subjects showing more than 50 per cent alpha waves (high vigilance group). Subjects of the latter group significantly overestimated time intervals in comparison to the former group. These results provide support for our hypothesis that states of vigilance detected by EEG directly influence time judgement.

Another explanation for the results of our study concerning the re-tapping speed might also be associated with altering internal clock speed. Although previous time estimation studies are based on the theory that the internal clock accelerates in more alert states and decelerates in drowsy states, this conclusion can not be drawn in every experimental paradigm. Due to a lacking observability of the speed of the internal clock, it can not be assumed with reasonable certainty that it indeed decelerates during drowsy states and accelerates during alert states.

In depression, several studies have reported an overestimation of length of time passing in depressed patients [44-48], whereas other studies have observed an underestimation of time [49] or did not find a definite alteration of time perception [50-52]. In contrast to several studies carried out with depressed patients, manic patients have only been investigated in three studies [44,49,53]. All three studies found an overestimation of time in time estimation tasks. During manic episodes, time perception might therefore be similar to that found in ADHD [54,55].

Both ADHD and mania are characterized by an unstable wakefulness regulation assessed by EEG measures of vigilance, ratings of sleepiness and deficits in sustained attention tasks. It has been postulated that in both mania and ADHD, this unstable wakefulness regulation represents a central pathogenetic factor leading to attention deficits and inducing hyperactive, impulsive and sensation-seeking behaviour as an autoregulatory attempt to stabilize wakefulness by increasing external stimulation [56].

Although our results clearly show significant correlations between vigilance states and time perception, several limitations should be pointed out. With 18 healthy subjects, the sample size was relatively small. Thus, it remains uncertain whether the findings are generalizable. This drawback becomes more evident for the analysis of vigilance sub-stages (A1, A2, A3, B1 and B2/3). In applying only a time production task, the obtained findings are not applicable to other time judgement paradigms, such as time estimation tasks. Investigations with large cohorts of patients and healthy controls with different standard tasks are required to validate the observed influence of the vigilance state on time perception. Another shortcoming of our study refers to the influence of emotion and personality on time perception [2,41]. As we did not control for individual differences, an interaction of present emotional arousal or stable personality and time perception can not be ruled out. Furthermore, we did not control for a regular sleep-wake schedule. Thus, the subjects might differ in their initial state of wakefulness, compliance and motivation in dependence on their sleep habits.

In general, our study indicates that EEG-based vigilance stages are associated with cognitive function, as e.g. time perception. Consequently, alterations of cognitive processing may be assessable by specific EEG-patterns and certain processing prototypes of EEG-vigilance stages. Therefore, the classification of EEG-vigilance stages via VIGALL may assist in identifying clinically relevant variations. Our findings regarding time perception during unstable wakefulness might also help to substantiate hypotheses regarding the role of wakefulness regulation in the pathophysiology of manic episodes. Nevertheless, further studies are needed to assess the general connection between the VIGALL-classified vigilance status and cognitive functioning.

## Competing interests

All authors declare not to have any conflict of interest including any financial, personal or other relationships with other people or organizations that could inappropriately influence, or be perceived to influence, their work. However, Prof. Himmerich received speaker honoraria from AstraZeneca, Lilly, Bristol-Myers Squibb and Servier, consulting fees from Bristol-Myers Squibb, and chemical substances for study support from AstraZeneca, Novartis and Wyeth. Prof. Hegerl received in the last three years honoraria as speaker or advisor from Lilly, Wyeth, Lundbeck, Bristol-Myers Squibb, Takeda and Sanofi-Aventis as well as a consultant for Nycomed.

## Authors' contributions

The presented work was carried out in collaboration between all authors. All authors were involved in drafting and revising critically the manuscript and approved the final version for publication. MT and UH defined the research theme and planned the conception of the study. MT and CS designed the experiment methods and acquired data. JM also acquired data. JM and HH made substantial contributions to the conception and design, analyzed the data, interpreted the results and wrote the paper. SO and AS directed the EEG recording methods and discussed analyses, interpretation and presentation. All authors read and approved the final manuscript.

## Disclosure statement

This work was supported by the Federal Ministry of Education and Research (BMBF), Germany, FKZ: 01EO1001.

## Supplementary Material

Additional file 1 **Table S1** EEG-based definition criteria of the VIGALL for the vigilance classification. Note: EEG-vigilance stages from full alertness to drowsiness are sub-classified (column 1) according to Bente [23] and Roth [24]. VIGALL classifies sub-stages based on EEG-power source estimates using sLORETA: A1 (occipital ROI power (α) > = parietal and frontal ROI power(α)), A2 (occipital ROI power (α) < parietal and frontal ROI power(α) and temporal and parietal ROI power(α) > = frontal ROI 1.5* power (α)), A3 (occipital ROI power (α) < parietal and frontal ROI power(α) and temporal and parietal ROI power(α) < frontal ROI 1.5* power (α)), B1 (power(α + δ + θ) in one $\text{ROI}=<7.5*10^{-6}\;\frac{\mu A^{2}}{mm^{4}}$ per data point), B2/3 (power(α + δ + θ) in one $\text{ROI}>7.5*10^{-6}\;\frac{\mu A^{2}}{mm^{4}}$ per data point). Column 3 describes the classification criteria of the EEG-vigilance main stages A and B. (JPEG 71 kb) Click here for file

Additional file 2 **Table S2** The assumed relationship between vigilance stages and the performed tapping speed. (JPEG 25 kb) Click here for file
