# Supplementary figures and images for: Time perception at different EEG-vigilance levels
Source: Behav Brain Funct. 2012 Sep 21;8:50. doi: 10.1186/1744-9081-8-50 (PMC3506441; doi:10.1186/1744-9081-8-50)

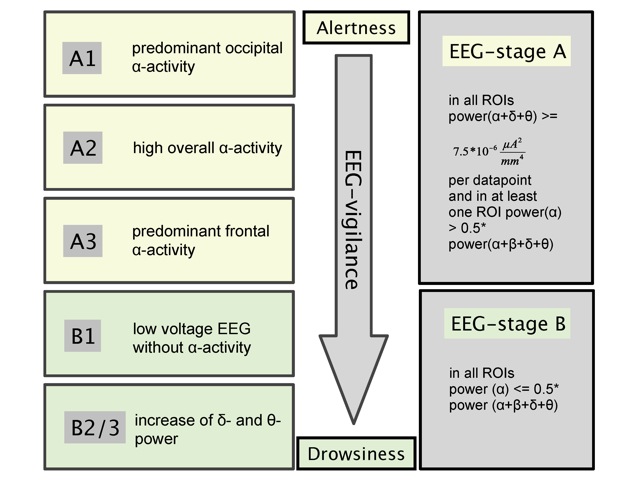

Supplement: Additional file 1 — Table S1 EEG-based definition criteria of the VIGALL for the vigilance classification. Note: EEG-vigilance stages from full alertness to drowsiness are sub-classified (column 1) according to Bente [23] and Roth [24]. VIGALL classifies sub-stages based on EEG-power source estimates using sLORETA: A1 (occipital ROI power (α) > = parietal and frontal ROI power(α)), A2 (occipital ROI power (α) < parietal and frontal ROI power(α) and temporal and parietal ROI power(α) > = frontal ROI 1.5* power (α)), A3 (occipital ROI power (α) < parietal and frontal ROI power(α) and temporal and parietal ROI power(α) < frontal ROI 1.5* power (α)), B1 (power(α + δ + θ) in one ROI=<7.5*10−6μA2mm4 per data point), B2/3 (power(α + δ + θ) in one ROI>7.5*10−6μA2mm4 per data point). Column 3 describes the classification criteria of the EEG-vigilance main stages A and B. (JPEG 71 kb) [file 1744-9081-8-50-S1.jpeg]

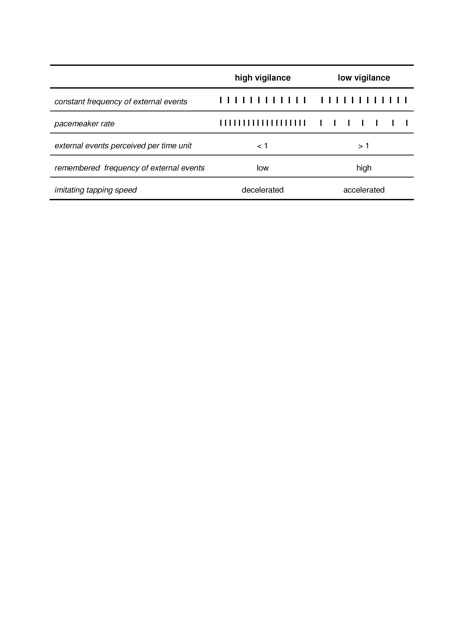

Supplement: Additional file 2 — Table S2 The assumed relationship between vigilance stages and the performed tapping speed. (JPEG 25 kb) [file 1744-9081-8-50-S2.jpeg]
